# Editorial: Sperm Differentiation and Spermatozoa Function: Mechanisms, Diagnostics, and Treatment

**DOI:** 10.3389/fcell.2020.00219

**Published:** 2020-04-07

**Authors:** Tomer Avidor-Reiss, Zhibing Zhang, Xin Zhiguo Li

**Affiliations:** ^1^Department of Biological Sciences, University of Toledo, Toledo, OH, United States; ^2^Department of Urology, College of Medicine and Life Sciences, University of Toledo, Toledo, OH, United States; ^3^Department of Physiology, Wayne State University, Detroit, MI, United States; ^4^Department of Obstetrics & Gynecology, Wayne State University, Detroit, MI, United States; ^5^Center for RNA Biology: From Genome to Therapeutics, Department of Biochemistry and Biophysics, University of Rochester Medical Center, Rochester, NY, United States; ^6^Department of Urology, University of Rochester Medical Center, Rochester, NY, United States

**Keywords:** spermatogenesis, sperm, capacitation, manchette, centriole, cilia, contraceptive pill, piRNA

## Introduction

One of the most remarkable processes in nature is the transformation of a generic round stem cell into a streamlined spermatozoon that fertilizes the egg, complements it, and activates the program that starts a new life. Sperm are unique because they undergo hyper evolution due to direct selection by sperm competition, resulting in many molecular, and structural invocations. This extraordinary biology affects virtually every process and structure of the sperm during spermatogenesis. The manchette reshapes the nucleus. The protamine repacks the DNA. New RNA granules form. The protein-based centrioles are remodeled. A flagellum with a unique configuration is formed. Membrane-bound organelles such as Golgi and ER are converted and reduced to acrosome and residual bodies. 80S ribosomes along with most of the cytoplasm are eliminated.

The spermatozoon continues to mature during its transport through the epididymis and female reproductive system. There, the spermatozoon gains motility, undergoes capacitation, and obtains epigenetic information. Hyperactivation and acrosome reaction allow it to fertilize eggs. Post-fertilization, the spermatozoon components complement the egg and activate embryo development.

All these unique properties of sperm are mediated by many sperm-specific proteins, making spermiogenesis an ideal target for male contraceptive pills. Abnormalities in sperm differentiation result in infertility, miscarriage, and congenital diseases, with a recent advance in transgenerational inheritance indicating that alterations in the sperm “epigenome” can impact the life-long wellness of offspring. Therefore, sperm dysfunction is a significant factor in men's infertility and farm animals' subfertility. Resolving this condition requires novel ideas at multiple levels to improve the diagnosis of sperm dysfunction and development of new treatments to complement *in vitro* fertilization (IVF) and intracytoplasmic sperm injection (ICSI). These also warrant the advancement of reproductive technologies such as sperm cryopreservation.

Below we present the 20 papers in this collection that address many of the critical aspects of sperm biology, medicine, and technology. We divided them into four categories: Unique Sperm Organelles, Spermatogenesis, Spermatozoon Maturation, and Sperm Contribution to the Zygote.

## Unique Sperm Organelles: Acrosome, Manchette, Atypical Centrioles, Migrating Cilia Transition Zone, and Fibrous Sheath

**The acrosome** is a specialized sperm organelle that contains digestive enzymes and covers the sperm head, which continues to surprise us with new findings (Zhang et al., 2020; Wang et al., 2020). It was proposed to be derived from the lysosome, or related organelle, or be a direct Golgi derivative (Hartree and Srivastava, 1965; Aguas and Pinto da Silva, 1985; Berruti et al., 2010). The review by Khawar in this collection provides a brief historical overview and highlights recent findings on acrosome biogenesis in mammals.

**The manchette** is a unique transient microtubular structure that shapes the sperm head and facilitates the development of the sperm neck and tail (Mendoza-Lujambio et al., 2002; Yang et al., 2018). A research paper in this collection by Tapia Contreras and Hoyer-Fender shows that the protein coiled-coil domain containing 42 (CCDC42) is a new centrosome protein that functions in the head-to-tail coupling apparatus (HTCA) and sperm tail formation.

**The centrioles** are an evolutionarily-conserved sub-cellular organelle that evolved to have unique properties in sperm cells (Avidor-Reiss, 2018; Avidor-Reiss and Turner, 2019). Each human spermatozoon contains two remodeled centrioles that it contributes to the zygote (Fishman et al., 2018). Most previous investigations into the role of mammalian centrioles during fertilization were completed in murine models. However, because mouse sperm and zygotes appear to lack centrioles, these studies provide information that is limited in its applicability to humans (Avidor-Reiss, 2018; Avidor-Reiss and Fishman, 2018). The review by Avidor-Reiss et al. in this collection comprehensively summarizes and updates the role of centrioles in human reproduction.

**The ciliary transition zone** is a gate separating the cilium from the cytoplasm via (Malicki and Avidor-Reiss, 2014). However, uniquely in the sperm, the transition zone migrates and separates the axoneme into two distinct compartments (Basiri et al., 2014; Avidor-Reiss and Leroux, 2015; Avidor-Reiss et al., 2017). Research papers in this collection by Persico et al. show that the microtubule-depolymerizing protein Kinesin-13 Klp10A is enriched in the ciliary transition zone and the base of the migrating transition zone of Drosophila melanogaster sperm. They suggest that Klp10A may be a core component of the ciliary transition zone.

**The fibrous sheath** is a unique sperm flagellum structure that is responsible for regulating signal transduction, metabolic pathways, and mechanical rigidity of the flagellum (Eddy, 2007; Mukai and Travis, 2012; Lindemann and Lesich, 2016). A-Kinase Anchor Protein 4 (AKAP4) is a major component of the fibrous sheath (Fang et al., 2019). A research paper in this collection by Nixon et al. found that AKAP4 is a target of oxidative stress caused by electrophilic aldehyde, 4-hydroxynonenal (4HNE) in sperm cells. These findings suggest that AKAP4 may be a biomarker of sperm quality, warranting the design of an antioxidant treatment for infertility.

## Spermatogenesis: Non-Hormonal Male Contraceptive Pill and Sperm Competition

**The non-hormonal male contraceptive pill** can be achieved by interfering with the many remarkable processes of spermatogenesis (Thirumalai and Page, 2019). The review by Kent et al. in this collection comprehensively summarizes recently discovered potential target genes for such a pill. They discuss novel contraceptive targets, new data for potential druggability, and possible effects from paralog proteins.

**Sperm competition** is a unique form of evolutionary selection that drives sperm innovation (van der Horst and Maree, 2014). Human sperm properties suggest that sperm have low-risk sperm competition as expected from a long history of male sexual dominance or monogamy. In naked mole-rats, only one male is reproductively active, and spermatogenesis is suppressed in the other males of the colony (O'Riain et al., 2000). This single male dominance example is a classic case of reducing sperm competition, leading to simplified, polymorphic, and slow-swimming spermatozoa (Van Der Horst et al., 2011). The research paper in this collection by van der Horst et al. show that lack of sperm competition also results in testicular structure and spermatogenesis degeneration.

## Spermatozoon Maturation in the Epididymis and Female Reproduction: Signaling Enzymes, Small RNAs, and Intracellular, and Extracellular Sperm pH

**Signaling enzymes** are central to the mechanism that regulates spermatozoon maturation since sperm is a transcriptionally silent cell (Freitas et al., 2017). Two papers address this subject in this collection. The review by Dey et al. recaps the contribution of four signaling enzymes that are present as specific isoforms only in placental mammals. Protein phosphatase PP1γ2, glycogen synthase kinase 3 (GSK3), calcium-regulated phosphatase calcineurin (PP2B), and protein kinase A (PKA) have critical roles in sperm maturation and hyperactivation. A research paper by Castillo et al. describes the quantitative proteomic profiling of capacitated and calcium activated spermatozoa. They found 36 proteins with significant changes in their relative abundance within these conditions and many that have post-translational modifications. These results contribute to our knowledge of the molecular basis of human fertilization.

**Small RNAs** such as PIWI-interacting RNAs (piRNAs), transfer RNA fragments (tRFs), microRNAs (miRNAs), and other non-coding RNAs are abundantly present in sperm and function in spermatogenesis and intergenerational epigenetic inheritance (Krawetz et al., 2011; Sharma et al., 2016; Sun et al., 2018; Perez and Lehner, 2019). The review by Sharma in this collection explores the mechanism of sperm small RNA remodeling during post-testicular maturation in the epididymis, and the potential role of this remodeling in intergenerational epigenetic inheritance.

**Membrane potential** is an essential feature during capacitation for sperm fertilization capability (López-González et al., 2014; Ritagliati et al., 2018). Two research papers in this collection develop methods to determine membrane potential. Molina et al. developed a technique based on flow cytometry and showed it can predict the fertilizing ability of human sperm. Baro Graf et al. developed a method based on potentiometric dye in a fluorometric assay. They both showed that the plasma membrane potential of capacitated sperm correlates with the sperm acrosome reaction.

**Intracellular and extracellular sperm pH** are important factors in sperm function as sperm encounter opposite PH conditions during its transport. Therefore, controlling sperm pH plays a crucial role in mammalian sperm physiology (Nishigaki et al., 2014). Two papers in this collection address this subject. A research paper by Chávez et al. established a robust imaging method that allows for the determination of absolute intracellular pH values in a single spermatozoon. The review by Touré specifies the current knowledge regarding PH regulation in sperm and its environment that is controlled by the distinct epithelia. They specifically discuss the role of solute carrier 26 (SLC26) proteins and their interaction with cystic fibrosis transmembrane conductance regulator channel (CFTR).

## Sperm Contribution to the Zygote: Egg Activation, Sperm Cryopreservation, Transient Sperm Starvation, and Intracytoplasmic Sperm Injection

**Egg activation** by sperm factor is essential to prevent polyspermy **(**Swann and Lai, 2016**)**. PLC-Zeta is a novel, testis-specific phospholipase C isoform that activates the egg post-fertilization (Swann et al., 2012). The review by Saleh et al. in this collection summarizes and updates the essential role of sperm-specific PLC-Zeta. However, PLC-Zeta may not be the only activation factor, and a potential “alternative” sperm factor may also be present.

**Sperm cryopreservation** is an essential technique for fertility management (Ezzati et al., 2020). However, the post-thaw viability of sperm differs among bulls—a research paper in this collection by Ugur et al. describes a multivariate and univariate analysis to identify potential freezability biomarkers. Their findings suggest that amino acids may have important roles in seminal plasma, although differences in amino acid concentration do not mediate this process.

**Transient sperm starvation** is a new and novel method to improve sperm performance, as described by the research paper in this collection by Navarrete et al.. This study discusses starvation increased hyperactivated motility, the ability to fertilize *in vitro*, and the production of pups in mice. Starvation also increased the fertility of a sub-fertile mouse and enhanced ICSI success in bovine. These findings raise the possibility that starvation may be used to improve assisted reproductive technologies in other mammalian species, including humans.

**Intracytoplasmic sperm injection (ICSI)** is a clinical treatment that introduces sperm to the oocyte independent of the sperm's ability to move and the two gametes' ability to fuse (Sánchez-Calabuig et al., 2014). Two papers in this collection address this subject. A research paper in this collection by Fernández-González et al. compared the ICSI success rate of spermatids from different regions of epididymis. They found that mice caput spermatozoa and caudal spermatozoa have similar potential to produce embryos and offspring by ICSI. A review in this collection by Oseguera-López et al. discusses novel techniques of sperm selection for improving IVF and ICSI outcomes. They describe the latest technologies focusing on those proven to improve sperm genetic integrity, fertilization capacity, embryo production *in vitro* survival, pregnancy, or delivery rates.

## Conclusion

Significant progress was made over the years in understanding the fundamental biology of the sperm. However, as evident from the studies described above, many aspects of this biology remain poorly understood. More studies are required to identify the molecular mechanisms that mediate and regulates spermatogenesis as well as spermatozoon maturation and capacitation. We need to understand better what controls sperm movement and its infraction with the female reproductive tract and the egg. A crucial future endeavor is identifying other essential contributions the sperm makes to the zygote in addition to the DNA. Gaining this information will guide future translational research towered the diagnosis and treatment of infertility, assisting the domesticated animal industry, the development of a male contraceptive pill, and improve the wellness of the next generation.

## Author Contributions

All authors listed have made a substantial, direct and intellectual contribution to the work, and approved it for publication.

### Conflict of Interest

The authors declare that the research was conducted in the absence of any commercial or financial relationships that could be construed as a potential conflict of interest.
